# Influence of the Sun's Rays in the Prodution of Organic Matter

**Published:** 1860-10

**Authors:** Chas. T. Jackson


### ARTICLE XVI.

*Influence of the Sun's Rays in the Production of Organic Matter.* By Chas. T. Jackson, M. D.

Plants alone possess the property of converting inorganic and mineral elements into organic substances, a power wholly denied to animals. They are enabled to effect this wonderful conversion through the influence of the solar rays, and chiefly by the decomposition of carbonic acid gas, the carbon being separated and combined with other elements, while the oxygen is given off and goes to form that portion of the atmosphere essential to the respiration of all animals. So rapid is the operation of the foliage of plants in abstracting carbon from carbonic acid of the air, that if we place a green and leafy bough of a tree in a glass globe, and place it in the sunshine, and then blow air through the globe, by means of a pair of bellows, the air, after passing over the foliage, will be found deprived of all its carbonic acid, and oxygen will have taken its place.—(Dumas.)

When we draw a breath of air into our lungs, and then exhale it, though it has been but a moment in the lungs, it will be found so charged with carbonic acid gas, as to extinguish a burning candle. This experiment is easily made by fixing a glass tube, inserted through a cork, into the top of a glass bell or receiver, open at the lower part, and placed in a vessel of water, and drawing the air from the receiver into the lungs, so that the water will rise and fill the vessel, and then breathing back the air into the receiver. Now if the cork is removed, and a lighted candle is lowered into the bell, it will be immediately extinguished. To show that foliage, in sunlight, will restore the respirable properties of the air, bend a leafy bough so that it shall pass under the edge of the bell and come in contact with the vitiated air, and expose the whole for a few minutes to sunlight. The carbonic acid will be decomposed, and the carbon being removed, oxygen will be left in the bell, and the candle being again applied will burn freely.

Water is not decomposed by the respiration of fishes, but only the air, dissolved in the water, goes to support their respiration, the proportion of air dissolved in water being, on the average, about $2\frac{1}{2}$ per cent. of its bulk.

Aquatic plants depend upon the small quantity of carbonic acid that is dissolved in water for the production of their carbonaceous tissues and juices, and they, like other plants, decompose carbonic acid through the influence of the solar rays, and give out oxygen. Thus plants and fishes aid each other, the one producing the proper respirable food for the other. Without aquatic plants, water would soon be unable to sustain the respiration of fishes; and hence in natural lakes and rivers, aquatic vegetation maintains the water in its proper condition for the respiration of these animals. So in well-balanced aquaria, the proper proportions of animal and vegetable life may be kept up, and, provided there is sufficient sun-light, no mechanical admixture of air is needed; for the plants will re-produce the oxygen from the carbonic acid exhaled from the fishes' gills.

The atmosphere is an ocean of mingled gases, chiefly nitrogen and oxygen, with a small proportion of carbonic acid, the proportions being, nitrogen 76·9, oxygen 23 per cent. by weight, while that of carbonic acid varies from three to sixteen thousandths parts. Aqueous vapor in variable proportions, is also dissolved in the air, the quantity depending on the temperature. Owing to the law of diffusion of gases, there is no separation of the heavier from the lighter by gravitation, and Gay Lussac found carbonic acid in the air he brought down in his balloon from a height of more than five miles, while Saussure ascertained its existence uniformly in the air over the highest peaks of the Alps. In large cities there is an accumulation of carbonic acid, owing to the want of free circulation of air; and the absence of an adequate amount of foliage for its removal; but it rarely accumulates in sufficient quantities to materially affect animal life. We live, then, at the bottom of a great atmospheric ocean, more than fifty miles deep.

The sun's rays penetrate readily through this atmosphere, and affect plants and animals on the earth's surface. From the dawn of creation—from the time when "God said let there be light, and there was light"—for myriads of ages, has the glorious orb of day been engaged in performing his beneficent work, and long before the creation of man the solar rays were busily employed in the preparation of the world for his advent.

Solar heat, absorbed by the wide-spreading ocean, at a time when scattered islands existed in the place of our present continents, warmed the waters, which so retained the heat as to give an almost tropical character to vegetation, even in climes far removed from the equator ; and the small area of land above the surface of the ocean radiated away into space but a small proportion of heat.—Hence, perhaps, the much wider extension of the tropical flora in ancient times, when the great coal formations were produced, and the remains of tropical animals in regions too far removed from the equator to allow of their existence in those regions now.

Some geologists think that the equalization of temperature, by circulating warm water, is adequate to account even for the fossil flora and fauna of Melville's Island and of the Siberian Coast, and also of the Arctic regions of America. Brogniart supposes that there was originally a larger proportion of carbonic acid in the air than now exists, and that under those favorable conditions of greater warmth and a humid climate of the oceanic islands, a rank vegetation grew and rapidly abstracted carbon from this gas, and converted it into those plants of which coal was formed.

By this operation ages of sunshine became converted into fossilized light and heat; for submerged plants were changed into bituminous coals, which at present supply us with light and heat, both of which, in the form of coal, were stored up long anterior to the creation of man.

This wonderful provision of coal, the source of most of the light and heat we enjoy in our dwellings—this accumulated and almost incalculable source of power concentrated in the bowels of the earth, was prepared by that being who created man, long before his coming, and thus the world was in the earliest stages fitted for the labors of civilized life, and the arts were provided with their most indispensable first materials, and the source of their greatest power.—*Boston Medical & Surgical Journal.*

